# Resistance Exercise Program Is Feasible and Effective in Improving Functional Strength in Post-COVID Survivors

**DOI:** 10.3390/jcm13061712

**Published:** 2024-03-16

**Authors:** Katarzyna Kaczmarczyk, Yogi Matharu, Patrycja Bobowik, Jan Gajewski, Agnieszka Maciejewska-Skrendo, Kornelia Kulig

**Affiliations:** 1 Faculty of Rehabilitation, Józef Piłsudski University of Physical Education in Warsaw, Marymoncka 34, 00-968 Warsaw, Poland; patrycja.bobowik@awf.edu.pl; 2 Division of Biokinesiology and Physical Therapy, University of Southern California, 1540 Alcazar St #155, Los Angeles, CA 90033, USA; matharu@pt.usc.edu (Y.M.); kulig@pt.usc.edu (K.K.); 3 Faculty of Physical Education, Jozef Piłsudski University of Physical Education in Warsaw, Marymoncka 34, 00-968 Warsaw, Poland; jan.gajewski@awf.edu.pl; 4 Faculty of Physical Culture, Gdansk University of Physical Education and Sport, 80-336 Gdansk, Poland; agnieszka.maciejewska-skrendo@awf.gda.pl; 5 Institute of Physical Culture Sciences, University of Szczecin, 70-453 Szczecin, Poland

**Keywords:** protocol, weakness, older adults, function, muscle

## Abstract

**Background**: Evidence suggests that COVID-19 infection can cause lasting health consequences. Multidisciplinary rehabilitation services have been recommended to reduce the sequalae. However, the effectiveness of physical exercise interventions remains insufficiently documented. The aim of this study was to develop and implement a specific and well-tolerated protocol-based intervention to reduce muscle weakness in older adults impacted by COVID-19. **Methods**: Forty-six older adults were randomized into intervention and control groups. Isometric and isokinetic strength assessments were conducted for selected muscle groups using a JBA Staniak^®^ torquemeter and Biodex System 3 dynamometer. Functional abilities were evaluated with the Time Up and Go test and Chair Stand Tests. **Results**: Men in the intervention group demonstrated a significant improvement in static conditions for knee flexors (KFs), trunk extensors (TEs) and trunk flexors (TFs) and in dynamic conditions for knee extensors (KEs). Women in the intervention group showed a significant improvement in static conditions for EFs, KFs, TEs and TFs and in dynamic conditions for a KE and a KF. The interaction GROUP × TESTING SESSION was significant for the Chair Test (s) and Chair Test (n). **Conclusions**: Our results demonstrate the effectiveness of a well-tolerated, protocol-based approach that can be used to diminish long-lasting functional deficits in post-COVID survivors.

## 1. Introduction

COVID-19 has affected more than 237 countries, and more than 517 million people have been infected across the globe, causing the deaths of more than 6 million people [1,2]. Vaccinations and the number of recovered individuals have weakened the effects of the virus; however, new variants continue to emerge. The understanding of acute symptoms and complications related to COVID-19 has been at the center of attention amongst the scientific community; however, emergent evidence has also shown persistent or new symptoms in survivors of COVID-19 [3]. WHO European regional data suggest 17 million people are affected [4]. Definitions of long COVID, referred to as Post-acute COVID-19 syndrome (PACS) or Post-acute Sequelae of SARS-CoV-2 (PASC), vary but are characterized by persistent symptoms and/or delayed or long-term complications persisting or beginning beyond four weeks from the symptom onset of a SARS-CoV-2 infection [5,6,7,8].

The WHO identifies more than 200 PACS/PASC-related symptoms that impact everyday activities. The five most common long-term symptoms are fatigue (58%), headache (44%), attention disorder (27%), hair loss (25%) and dyspnea (24%) [9]. Other persistent symptoms have been reported, including cough, chest pain, myalgia, joint pain, impaired mobility, cognitive impairment, olfactory and gustatory dysfunction, sleep disorders, depression, anxiety, post-traumatic stress disorder, gastrointestinal upset, rashes and palpitations [10,11,12,13,14,15]. As an international priority, the US has dedicated $1.15 billion for diagnosis and treatment research [16]. 

Muscle damage resulting from COVID-19 infection has been reported. Specifically, both cardiac and skeletal muscle tissues exhibit robust ACE2 (angiotensin-converting enzyme 2) and TMPRSS2 (transmembrane serine protease 2) expression, indicating a potential vulnerability to SARS-CoV-2 infection in muscles. This damage results from inflammatory effects, a cytokine storm and muscle catabolism [17,18,19]. Consequently, damage to muscles will result in a decrease in muscle strength and may contribute to pain. In previous studies, we demonstrated a significantly lower level of muscle strength in women after recovering from COVID-19 compared to age-matched healthy individuals [20].

Moreover, even without COVID-19 infection, in the 6th decade of life, an accelerated, non-linear decrease in muscle strength has been reported (up to 15%), and by the 8th decade, this strength loss may be up to 30% in healthy elderly people [21]. In people with a less active lifestyle, these age-related changes manifest earlier [22]. This natural age-related loss is compounded by COVID-19 infection and pandemic protocols causing decreased socialization and work/exercise outside of the home. Once infected with COVID 19, being prescribed rest to minimize metabolic demands and channel resources towards the recovery process may have accelerated a functional decline in elderly patients. As a point of reference, Kortebein et al. (2008) found a substantial loss in muscle strength and power (knee extension *p* = 0.004, knee flexion *p* = 0.003 and stair ascent power *p* = 0.01) after 10 days of bed rest in healthy elderly people (60–85 years old) [23]. 

To reduce the consequences of COVID-19 infection, the NICE guideline [24] and American consensus [25] recommend integrated multidisciplinary rehabilitation services for individuals with long-term effects of COVID-19. However, the effectiveness of exercise interventions for post-COVID patients remains insufficiently documented. Most reports have concerned the effects of pulmonary rehabilitation, where the study groups were compared to no rehabilitation/drug-only intervention [26,27,28]. 

Previous studies have suggested that resistance training is both safe and highly effective in combatting strength loss and declining functional capacity in the healthy elderly [29,30,31]. It has not been established that these treatments are safe to implement or effective in individuals with PACS/PASC. Furthermore, the high volume of affected individuals exceeds the availability of rehabilitation specialists to create individual programs.

Therefore, the aim of this study was to develop and implement a specific, early and well-tolerated protocol-based intervention to reduce muscle weakness in older adults impacted by COVID-19. The secondary aim was to test the hypothesis that resistance training will improve muscle strength in post-COVID survivors and that the strength gains will be accompanied by improved function. 

## 2. Materials and Methods

### 2.1. Subjects

Participants were recruited from nursing homes, primary health care facilities and a University of the Third Age program, using the social media accounts of a local university, and from surrounding communities using posters, leaflets and videos from January to March 2023. The inclusion criteria were as follows: both sexes, aged 65 and older, having had a positive RT-PCR test and/or a positive result in a test for antibodies against the SARS-CoV-2 coronavirus in the 3–12 months prior to the commencement of the study and reporting one or more of the post-COVID signs and symptoms, such as fatigue, muscle weakness, dizziness, headache, memory and concentration disorders, exercise intolerance or depression. Before initiating the rehabilitation program, participants were screened by a physician, and the exacerbation of post-exercise symptoms was assessed based on a questionnaire [32] and an orthostatic test [33]. People aged less than 65, with active cardiac disease, oxygen desaturation below 95% for more than 1 min, dysfunctions of the autonomic nervous system (orthostatic intolerance) and serious health conditions such as cancer were excluded from this study. 

**Figure 1:**
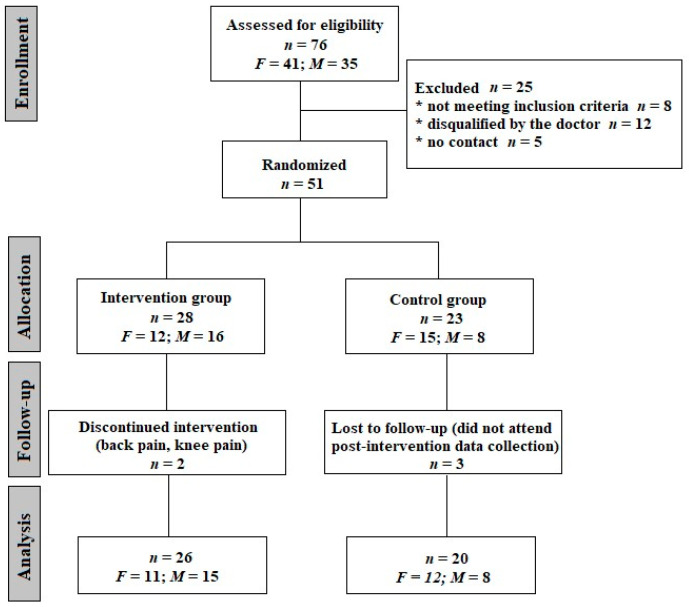
CONSORT flow diagram of the progress though the phases of parallel trial in two groups. *F*—females; *M*—males.

It is worth mentioning that 92% of the respondents had been vaccinated with at least one dose of the anti-SARS-CoV-2 vaccine, and only 27% of the study participants had become unwell before the vaccine. The average time from the onset of the disease in people qualified according to the inclusion criteria was 9 months, and 33% described the infection as mild, 51% as moderate, 10% as severe and 6% as very severe. A post-infection interview regarding the recall of signs and symptoms was conducted. The symptoms reported by the participants included dizziness and equilibrium disorders (reported by 55% of respondents), perceived muscle weakness (35.3%), exercise intolerance (31.3%) and memory and concentration symptoms (19.6%). Other, less frequently reported symptoms included cough, dyspnea, libido deterioration, insomnia and deterioration of taste and smell. After meeting the inclusion criteria and passing the medical screening, participants were allocated into one of two groups: an intervention group, which received resistance training, and a control group, whose members were advised to retain their activity level as usual. Allocation to the groups was carried out randomly using an Excel random number generator. Finally, 46 people completed the study protocol, including the pre- and post-intervention testing conducted in a Central Laboratory at the Józef Pilsudski University of Physical Education in Warsaw. The consort diagram is presented in Figure 1.

The data from the 46 post-COVID seniors were analyzed. Anthropometric characteristics of the tested groups at baseline are shown in Table 1. No significant differences were found between the intervention and control groups in anthropometric parameters, except for the age in the men’s groups (*p* < 0.05). The necessary minimum total number of subjects (n = 40) was obtained using the G*Power program assuming detection of medium-sized effects (η^2^ = 0.06) at a significance level of a = 0.05 and statistical power of 0.85.

### 2.2. Methods

#### 2.2.1. Muscle Strength Tested in Static Conditions

Strength, defined as the ability to produce force, was measured in two conditions: static and dynamic. The results of the testing in static conditions will be referred to as the moment of force. All the measurements were performed after the subject was familiarized with the measurement protocol and after a warm-up. Participants were instructed to exert maximal effort while pushing a bar or testing apparatus for 3 s. The subjects performed 3 repetitions with one minute of rest between the measurements. The highest value (peak) was used for the statistical analysis. Finally, the absolute [N·m] and relative to body mass [N·m·kg^−1^] values of the following parameters of the strength were taken for the analysis: elbow flexors (EFs), knee flexors (KFs), knee extensors (KEs), trunk flexors (TFs) and trunk extensors (TEs).

**Figure 2:**
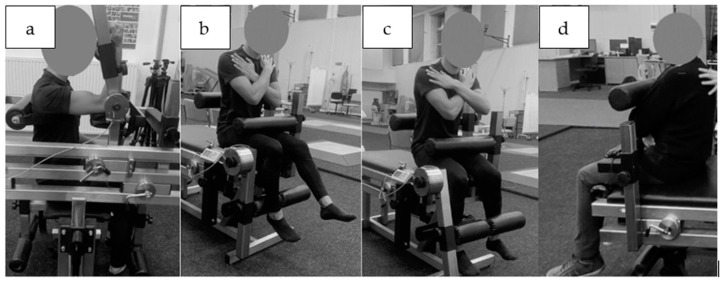
Muscle strength testing position in static conditions, (**a**) elbow flexors, (**b**) knee extensors, (**c**) knee flexors, (**d**) trunk flexors.

The peak moment of force values for each muscle group were extracted for further analysis, namely, the flexors and extensors of the knee, trunk, and flexors only of the elbow. The measurements were taken on a custom-made device (“JBA”, Zbigniew Staniak, Zgorzelec, Poland)—type LR1-P (upper extremities) and TBK2-PM (lower extremities and trunk). Technical specifications of the measuring device are as follows: torque meter: strain gauge head—measuring range for 0 to 500 N·m for LR1-P and 0–2000 N·m for TBK2-PM, the relative error in the strain gauge bridge amounts to 1.0%. Measurements of muscle torque were performed according to generally accepted principles [34]. The dominant upper extremity’s forearm flexors were tested seated, the angle at the shoulder joint was 90 degrees of flexion and the supported forearm was perpendicular to the arm. The trunk was stabilized by a belt (Figure 2a). 

The knee flexor and extensor torque of the dominant limb were also tested in the sitting position, with the hip and knee joints at 90° of flexion. The subjects were stabilized by a close-fitting roller at the level of the anterior iliac spine at the proximal part of the thigh and posteriorly at the lumbar spine. The upper limbs were crossed over the chest (Figure 2b,c). The trunk flexors and extensors were tested in the sitting position and the estimated axis of rotation at the hip coincided with the axis of rotation of the torque meter lever. The participants were also stabilized by a roller at the level of the proximal part of the thigh with the upper limbs crossed over the chest (Figure 2d).

#### 2.2.2. Isokinetic Strength Evaluation

**Figure 3:**
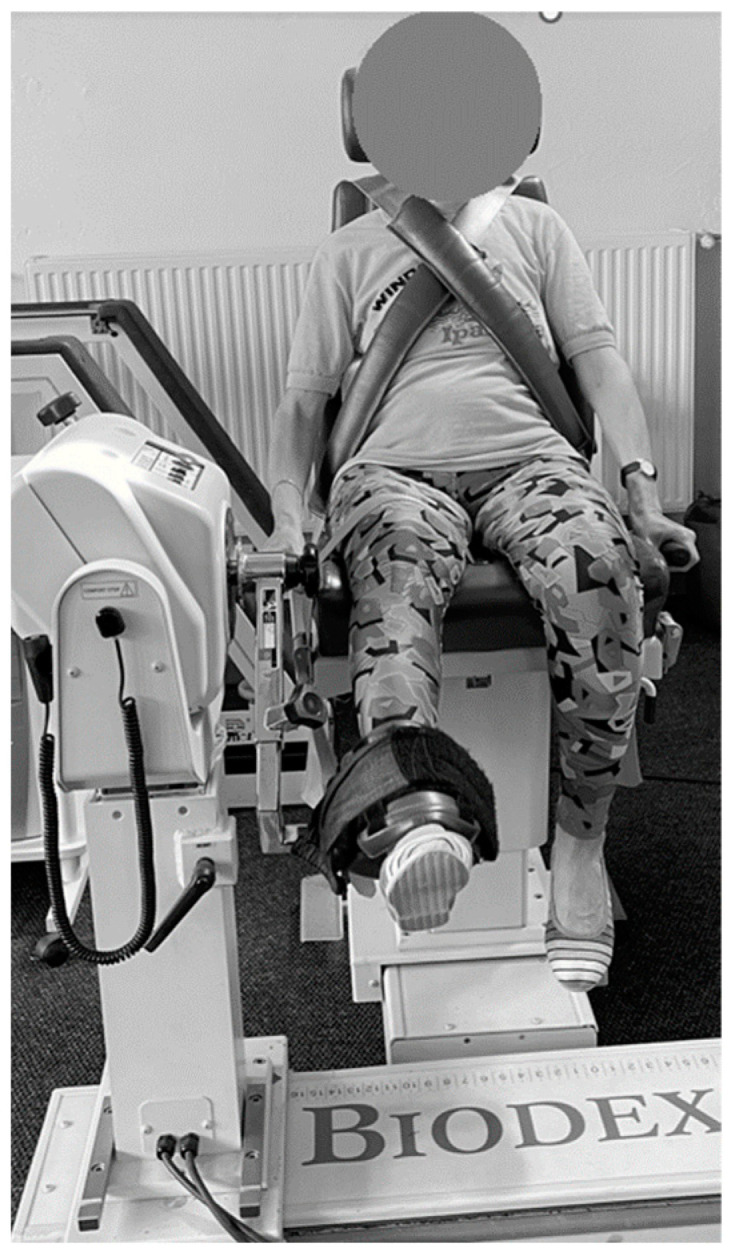
Muscle strength testing position in a dynamic condition—isokinetic knee extension and flexion at 60°/s (5 repetitions) and 180°/s (10 repetitions).

The dynamic strength evaluation was conducted using an isokinetic dynamometer. The Biodex System 3 dynamometer (Biodex Medical Systems, Inc., Shirley, NY, USA) was used for the isokinetic strength assessments. Only the dominant leg was tested. Isokinetic knee extension and flexion pattern at 60°/s (5 repetitions) and 180°/s (10 repetitions) were tested. A 90-s rest time was provided between sets. Subjects were seated in the chair of the dynamometer and stabilized by belts around their trunk, pelvis and mid-thigh of the test leg. Hip flexion was set at 85° and the dynamometer axis was aligned to the knee’s anatomical axis of rotation. The ankle pad was positioned just above the medial malleolus (Figure 3). 

The range of motion (ROM) was limited to between 90° and 0° of flexion. The extension ROM for each participant was defined in accordance with their individual limits. Participants were instructed to exert maximal effort during each test while verbal encouragement and visual feedback were provided. The following parameters were analyzed: Peak Torque E60 [N·m], Peak TQ/BW (%) E60 [N·m·kg^−1^], Peak Torque F60 [N·m], Peak TQ/BW (%) F60 [N·m·kg^−1^], Peak Torque E180 [N·m], Peak TQ/BW (%) E180 [N·m·kg^−1^], Peak Torque F180 [N·m] and Peak TQ/BW (%) F180 [N·m·kg^−1^].

#### 2.2.3. Functional Tests

##### Time Up and Go test (TUG)

The TUG test required participants to start from a seated position in a standardized chair, stand up and walk for 2.44 m, walk around a cone, return to the chair and get back into the starting position. The time was measured in seconds. 

##### Chair Stand Test

- CS-30 scores—participants were instructed to complete sit-to-stand trials using a 40 cm high seat without using their arms as many times as possible in 30 s [35]. The number of stands was recorded.
- 5STS—participants were asked to sit on a 40 cm high seat without using their arms and then stand repeatedly for five times as quickly as possible [36]. The time was recorded.

### 2.3. Intervention

Resistive training (RT) aimed at improving muscle strength was conducted in the Zdrofit Gym Warsaw-Bielany twice a week, for 60 min per session and for 8 weeks, according to the recommendations of World Physiotherapy (World Physiotherapy, 2021 and NICE, 2020) [24]. The protocol was developed by a team of physicians, specialists in physiotherapy and resistance training coaches. Before every session, participants’ heart rate, blood pressure and oxygen saturation were measured. If they had a blood pressure of >160/100 mmHg or a heart rate (HR) > 100 or <50 beats per minute, participants were not allowed to do exercises during that session. 

The first training session included the determination of the 1 Repetition Maximum (1RM) on each exercise. Therefore, all the subjects performed 4–5 trials with increasing load and rest periods between the trials and with 3 min of passive recovery. The goal was to perform 3–5 repetitions with maximum load. The subjects were instructed to perform the exercise at a comfortable pace. The 1RM was calculated according to the formula developed by Brzycki (1993) [37]. 

**Figure 4:**
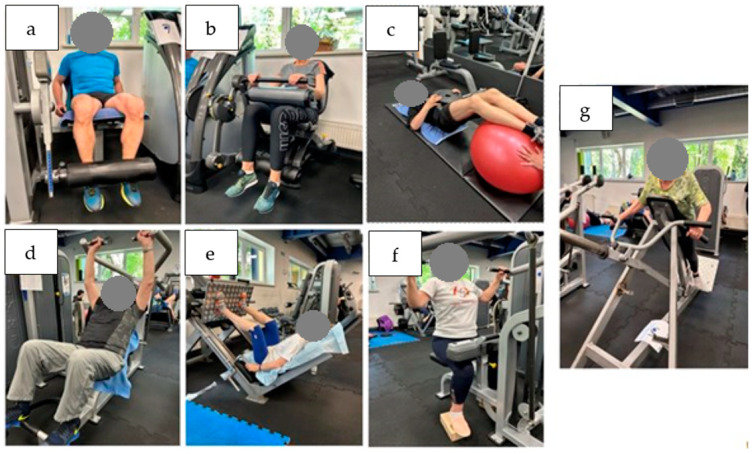
Exercises in each session: (**a**) leg extension, (**b**) leg curl, (**c**) trunk crunch, (**d**) incline bench press, (**e**) 45 degrees leg press, (**f**) latissimus pull-down, (**g**) T-Bar row.

Each training session aimed to reach an exercise intensity of 70% of 1RM and consisted of three sets of 12 repetitions on each exercise (incline bench press, 45 degree leg press, latissimus pull-down, trunk crunch, T-Bar row, leg extension and leg curl) (Figure 4).

The rest periods between the sets included a 2 min passive recovery. Before performing each training session, the subjects performed a 15 min general warm-up on an Orbitrek or treadmill with individual intensity of 60–65% HRmax. The training loads during the training sessions were increased individually by 5 kg when a subject completed all the repetitions during an exercise.

### 2.4. Statistical Analysis

Statistical analysis was performed using STATISTICA 14.0 (TIBCO Software Inc., Palo Alto, CA, USA (2020) and the Data Science Workbench, version 14. http://tibco.com, accessed on 1 February 2023). The normality of the distributions of the study variables in the groups was tested using the Shapiro–Wilk test. Comparisons of the means were made using analysis of variance for the repeated measures. GROUP (Control, Intervention) and SEX (Men, Women) were used as fixed factors. The repeated measures factor TESTING SESSION took two values: Before and After. Due to the primary aim of the study, it was assumed that the different responses of the study groups would be described by the GROUP × TESTING SESSION interaction. Detailed comparisons were carried out using the Tukey post hoc test. In the case of variables not meeting the condition of normality of distributions, the Mann–Whitney U test was used for comparisons between groups. Changes in variables before and after the intervention were assessed using the Wilcoxon test. For these variables, the response to the intervention was assessed by comparing the increments of the study variables in the two groups (Intervention, Control) using the Mann–Whitney U test. 

Effect sizes were assessed using partial eta square (ANOVA), Glass’s rank-biserial correlation coefficient (Mann–Whitney test) and equivalent correlation coefficient (Wilcoxon test). A significance level α = 0.05 was adopted. 

## 3. Results

The results for the seniors in the intervention group who did not miss more than three sessions were taken for analysis. Two subjects were lost due to unrelated musculoskeletal pain (lower back, knee pain). The average attendance rate was 93% (80–100%). 

Post-infection interviews regarding the recall of signs and symptoms were conducted in the intervention group for comparison with the results before training. The frequency of post-COVID-19 symptoms before and after the RT are shown in the table below (Table 2). 

The results of two testing sessions (pre- and post-intervention tests) of maximal muscle torque relative to body mass values [N·m·kg^−1^] in static conditions are shown in Table 3. In the intervention group, in both the females and males, an increase in all the parameters was observed. The largest increase was found in trunk flexion (18.9%) and extension (22%) in the group of active men (*p* < 0.001). In turn, in the group of active women, the most significant improvements were noticed in the elbow flexion (17%), knee flexion (20.2%) and trunk extension (23.3) (*p* < 0.01). 

In the control group, a decrease in most of the parameter values expressed relative to body mass [N·m·kg^−1^] was observed in the group of men, in contrast to the women, who had improved their results, but not significantly. To assess the effects of the intervention, GROUP × TESTING SESSION (pre- and post-intervention tests) interactions were considered. The interaction was significant for the relative maximal muscle torque of all the measured muscle groups (*p* < 0.001) (Table 3). 

Statistical analysis revealed significant improvement in the knee extension isokinetic torque (60°/s) in the absolute values and those normalized to body mass (*p* < 0.05 and *p* < 0.01, respectively) and in the knee flexion isokinetic torque (60°/s) (*p* < 0.05) in the women’s group after intervention. In the intervention group of men, the significant improvement was observed in the knee extension isokinetic torque (180°/s) normalized to body mass (*p* < 0.05). However, due to the increase in the isokinetic torque in both groups (control and intervention), the effects of the intervention GROUP × TESTING SESSION (pre- and post-intervention tests) interactions were not found (Table 4 and Table 5). The results of the flexors/extensors ratio are presented in Table 6. The values of the F/E ratio increased in the intervention group; however, the effects of the intervention GROUP × TESTING SESSION (pre- and post-intervention tests) interactions were not observed.

The results of the three functional tests are presented in Table 7. In the intervention group, both in men and women, a significant improvement was observed in the Chair Test (s) (*p* < 0.001 and *p* < 0.05, respectively) and the Chair Test (n) (*p* < 0.01 in both groups). The interaction GROUP × TESTING SESSION (pre-and post-intervention tests) was significant for the Chair Test (s) (F_1,42_ = 8.49, *p* < 0.01, η^2^ = 0.68) and the Chair Test (n) (Z = 4.65, *p* < 0.001, R = −0.806).

## 4. Discussion

Our results show that there was improvement in strength and function utilizing a protocol-based intervention of resistance training in elderly adults post-COVID-19 infection. The specific protocol used was short enough to be plausible in a clinical setting and was well tolerated, and the patient was able to adhere to the program as evidenced by the low drop-out rate. 

The seven equipment-based progressive resistive exercises targeted three body regions, namely, the upper extremity, lower extremity and trunk. For testing purposes, the sagittal plane isometric strength of the elbow, knee and trunk was selected. Additionally, the knee extensors and flexors were tested isokinetically at 60 degrees/seconds and 180 degrees/seconds. 

Elbow flexor strength is among the most tested muscle groups of the upper extremity. This is partially due to the ease and reliability of the testing procedure. A recent study by Rodriguez-Rodriguez determined that elbow flexor power, tested in a seated position, is strongly related to the well-established whole body functional test used with older adults [38]. In our study, elbow flexor strength performance, tested isometrically, was a proxy for overall upper extremity physical performance. At baseline, the average, normalized to body weight elbow flexor strength was 0.75 ± 0.14 for men and 0.49 ± 0.16 for women. These values were higher than those estimated from a subgroup of 178 healthy adults for male subjects (0.59) and female (=0.38) [39]. In our study, a post hoc analysis determined an improved strength in women (from 0.49 ± 0.16 to 0.59 ± 0.13), but not in men. These comparisons suggest that the subjects in our study, especially the women, were not overly weak and were able to improve their elbow flexor strength.

Knee flexors and extensors are the primary muscle groups selected for strength testing in the lower extremity. The seated position and ease of thigh and trunk stabilization, in addition to the known susceptibility to age-related muscle loss, make this test the first choice for lower extremity strength testing [40]. At entry into this study, the normalized isometric knee flexor strength (Nm/kg) in men and women, was higher than that reported by Sarabon et al., who found 1.08 versus 0.79 for men and 0.75 versus 0.64 for women [40]. Our intervention improved knee flexor strength in men from 1.08 ± 0.31 to 1.21 ± 0.28 and 0.75 ± 0.22 to 0.94 ± 0.28 for women. It is worth noting the surprising similarity of the pre- and post-intervention test 8-week data for the control group (mean difference for men 0.03 and null difference for women). Indirectly, the consistent data for the control group speak to the reliability of knee flexor strength measurements in our study. Isometric knee extensor strength was lower than in a group of healthy older adults [41] and higher than in a group of institutionalized but independent women older than 70 years of age [42]. Although the averaged body weight normalized values improved in the intervention group, our GROUP × TESTING SESSION design did not show differences in a post hoc analysis.

Since its inception in the 1960s, isokinetic knee torque performance has been used extensively in characterization and intervention studies providing wide-ranging comparative data. The touted benefits of isokinetic strength testing are the dynamic testing of strength at a preset constant velocity, allowing the torque to be measured reliably throughout the range of the joint’s motion. In this study, we selected the two most tested velocities, 60 and 180 degrees/seconds. The body weight normalized peak torque at 60 degrees/seconds was within the values reported for testing in older adults [43,44]. Our 8-week strengthening intervention did not result in altered peak torques at either velocity. This is intriguing, since the exercise program was equipment-based and dynamic, and the isometric strength discussed above improved. It is possible that the moving resistance arm during the isokinetic testing provides for a secondary cognitive demand, or perhaps fear resulted in a lower torque output.

Although trunk strength testing is less commonly implemented than elbow and knee testing, this metric provides a valuable quantitative characterization of muscle performance in the largest segment of body mass. At entry into this study, our cohort’s average isometric trunk extensor strength, normalized to body weight, was within the range of values reported by Keinbacher et al. on a population of older adults aged 50 to 90 years (average age 67.2 years old) with a history of lower back pain [45]. In the current study, the 8-weeks exercise program significantly increased the normalized trunk flexor strength from 1.84 Nm/kg to 2.27 Nm/kg for men and from 1.14 Nm/kg to 1.46 Nm/kg for women.

Common daily tasks such as stand-to-sit and sit-to-stand, as well as walking initiation and termination, have become the basis of widely used standardized functional tests in clinical trials and in the clinic. This study chose to use three functional tests: Time Up and Go, repeated sit-to-stand, number of sit-to-stand moves performed in 30 s.

The TUG test is performed in the following sequence: subjects start in the seated position, stand up, walk 2.44 m and return to a seated position as fast as possible. The results are reported in seconds. In addition to functional mobility and gait, the Time Up and Go (TUG) test assesses non-vestibular and vestibular aspects of balance relevant for post-COVID symptoms. This test requires controlling whole body acceleration in the vertical and fore–aft directions. The older adults in our study performed the test within the ranges reported in the literature (5.1 to 9.0 s) [46,47,48,49,50]. There was no statistically significant improvement in performance, since the results at entry into the study were typical for this age group. The repeated sit-to-stand test is performed 5 times. The results are reported in seconds. The test requires controlling vertical acceleration of the center of mass. The average baseline values in the current study ranged between 8.78 ± 1.98 and 10.78 ± 4.45 s, which was shorter than the average values found for older adults in a large study (5352 participants) by Ostchega et al., namely, 13.11 ± 0.19 for men and 14.05 ± 0.2 for women [51]. Both the male and female subjects in our study performed the test faster than this, but the participants in the intervention study showed significant improvement. Finally, to gain insights into fast transition movements, this study chose to use the 30 s sit-to-stand test. The outcome of this test is the number of completed repetitions. At entry into this study, the number of repetitions ranged between 13 and 22. Both intervention groups improved significantly, with males increasing from 15 to 24 and females from 18 to 25 sit-to-stand repetitions in 30 s. This improvement is similar to the one reported by Sato et al. testing the effects of exercise games (rising from 17 to 24 repetition in a group of women aged 70 ± 5.4 years) [52].

There are several limitations within this study. The first is the small number of subjects in the study. While larger numbers would be preferred, the size is appropriate considering that this is one of the first studies utilizing an active exercise program following the end of the public health emergency. It was important to establish the safety and effectiveness of the protocol before increasing the size of the cohort. The second limitation is that the participants in the intervention group were already functioning at a reasonably high level. Once again, establishing efficacy in a higher-functioning group was an important first step. It is likely that a group functioning at a lower level would have achieved even greater gains. Another limitation is that we were not able to systematically track symptom improvement within the control or intervention group. Future studies will expand on this work by using a larger cohort and assessing the impact on a cluster of symptoms. The last limitation is that we used a community gym equipped with resistance exercise machines. That resource may not be available in every community, and modifying the exercise program with body weight resistance may be worthwhile. If specialist equipment is not available, other types of resistance utilizing dumbells or resistance bands may yield similar results.

## 5. Conclusions

The COVID-19 pandemic had an immediate and profound economic and health impact across the world. Obvious impacts on health included illness, hospitalization and death. However, we are now realizing that the disease also created a long-term impact that will last for generations. Many individuals are suffering from poorly understood symptoms of long COVID and many members of our society have not recovered from the effects of decreased physical activity and increased sitting time necessitated by online meetings and work.

The pandemic’s impacts on bodily systems require rehabilitation. While we cannot yet address all the problems, we can address the strength deficits that impact function and long-term health. This will lower the burden of care, lower the risk of falls, reduce the assistance needed, increase independence and improve quality of life.

As generalized fitness programs may not be appropriate for people who have been infected with COVID-19 or suffer from other chronic conditions, there has been a significant demand on medical professionals. Unfortunately, these specialists are unavailable because of the extreme backlog of deferred care that will last for many years. It is critical that a program that can be individualized based on relevant criteria is developed and demonstrated to be effective and well tolerated.

Our results demonstrate the effectiveness of a well-tolerated, protocol-based approach that can be used by professionals to diminish the long-lasting functional deficits following COVID-19 infection in post-COVID survivors.

## Figures and Tables

**Table 1:** Anthropometric characteristics of the tested groups at baseline.

	Intervention (f = 11, m = 15)	Control (f = 12, m = 8)
Female		
Age (years)	69.3 ± 5.2	73.3 ± 7.4
Body mass (kg)	65.3 ± 10.5	72.6 ± 12.1
Height (m)	163.1 ± 7.6	160.9 ± 5.7
BMI (kg/m^2^)	24.5 ± 4.8	28.1 ± 3.3
Male		
Age (years)	69.5 ± 4.8	75.6 ± 7.1 *
Body mass (kg)	87.7 ± 15.1	89.5 ± 20.3
Height (m)	176.7 ± 6.9	179.0 ± 6.1
BMI (kg/m^2^)	27.86 ± 3.4	27.07 ± 5.4

Different than in Intervention group: *—*p* < 0.05.

**Table 2:** Self-reported percentage of common post-COVID-19 symptoms in an intervention group before and after training.

Post-COVID-19 Symptom	Dizziness and Equilibrium Disorders	Perceived Muscle Weakness	Exercise Intolerance	Memory and Concentration Symptoms
Before intervention	46.2%	38.5%	38.5%	15.3%
After intervention	15.4% *	7.6% **	3.8% **	7.6%

Different than pre-intervention: *—*p* < 0.05, **—*p* < 0.01.

**Table 3:** Absolute values of maximal muscle torque at the elbow, knee and trunk in static conditions normalized to body mass.

		GROUP						
		Control		Intervention				
	TESTING SESSION					GROUP × TESTING SESSION		
		Male (n = 8)	Female (n = 12)	Male (n = 15)	Female (n = 11)	F_1,42_	*p*	η^2^
EF/m (N·m·kg^−1^)	Before	0.71 ± 0.19	0.42 ± 0.10	0.75 ± 0.14	0.49 ± 0.16	17.41	0.0001	0.293
	After	0.62 ± 0.14	0.45 ± 0.09	0.78 ± 0.13	0.59 ± 0.13 **			
KF/m (N·m·kg^−1^)	Before	0.94 ± 0.22	0.69 ± 0.21	1.08 ± 0.31	0.75 ± 0.22	10.15	0.0027	0.195
	After	0.97 ± 0.27	0.69 ± 0.18	1.21 ± 0.28 *	0.94 ± 0.28 **			
KE/m (N·m·kg^−1^)	Before	1.55 ± 0.39	1.23 ± 0.27	1.84 ± 0.54	1.43 ± 0.43	11.35	0.0016	0.213
	After	1.26 ± 0.38	1.28 ± 0.22	2.00 ± 0.46	1.57 ± 0.45			
TF/m (N·m·kg^−1^)	Before	1.51 ± 0.40	1.05 ± 0.31	1.84 ± 0.52	1.14 ± 0.33	14.21	0.0005	0.253
	After	1.44 ± 0.38	1.13 ± 0.42	2.27 ± 0.67 ***	1.46 ± 0.34 *			
TE/m (N·m·kg^−1^)	Before	2.65 ± 0.77	1.64 ± 0.69	3.20 ± 1.08	2.64 ± 1.23	15.75	0.0003	0.273
	After	2.48 ± 0.76	1.84 ± 0.82	4.10 ± 0.81 ***	3.44 ± 1.30 **			

Difference in pre- and post-intervention tests: *—*p* < 0.05, **—*p* < 0.01, ***—*p* < 0.001. EF—elbow flexor, KF—knee flexor, KE—knee extensor, TF—trunk flexor and TE—trunk extensor, m—body mass.

**Table 4:** Knee extension and flexion isokinetic torque (60°/s).

		GROUP						
		Control		Intervention				
	TESTING SESSION					GROUP × TESTING SESSION		
		Male (n = 8)	Female (n = 12)	Male (n = 15)	Female (n = 11)	F_1,42_	*p*	η^2^
Peak Torque E60 (N·m)	Before	109.3 ± 34.8	78.5 ± 20.0	143.5 ± 39.4	84.1 ± 15.8	1.06	0.3095	0.025
	After	120.5 ± 24.5	80.0 ± 19.0	160.0 ± 37.2 *	90.9 ± 18.6			
Peak Torque/m E60 (N·m·kg^−1^)	Before	1.29 ± 0.59	1.10 ± 0.30	1.65 ± 0.36	1.31 ± 0.28	1.11	0.2982	0.026
	After	1.47 ± 0.55	1.11 ± 0.29	1.86 ± 0.40 **	1.43 ± 0.31			
Peak Torque F60 (N·m)	Before	68.1 ± 29.4	45.4 ± 19.6	76.4 ± 31.1	46.4 ± 13.5	3.73	0.0601	0.082
	After	69.2 ± 23.0	47.2 ± 14.1	88.3 ± 27.2 *	54.3 ± 13.6			
Peak Torque/m F60 (N·m·kg^−1^)	Before	0.78 ± 0.36	0.64 ± 0.30	0.88 ± 0.34	0.74 ± 0.26	3.30	0.0766	0.073
	After	0.83 ± 0.37	0.65 ± 0.20	1.02 ± 0.28	0.86 ± 0.25			

Different than in pre-intervention test: *—*p* < 0.05, **—*p* < 0.01 Peak Torque E60—peak torque of knee extension at 60°/s, Peak Torque/m E60—peak torque of knee extension at 60°/s normalized to body mass, Peak Torque F60—peak torque of knee flexion at 60°/s, Peak Torque/m F60—peak torque of knee flexion at 60°/s normalized to body mass.

**Table 5:** Knee extension and flexion isokinetic torque (180°/s).

		GROUP						
		Control		Intervention				
	TESTING SESSION					GROUP × TESTING SESSION		
		Male (n = 8)	Female (n = 12)	Male (n = 15)	Female (n = 11)	F_1,42_	*p*	η^2^
Peak Torque E180 (N·m)	Before	65.08 ± 13.36	45.00 ± 19.29	88.32 ± 25.72	48.33 ± 14.62	0.16	0.6870	0.004
	After	78.15 ± 14.81	53.16 ± 14.07	96.62 ± 20.05	58.12 ± 12.33			
Peak Torque/m E180 (N·m·kg^−1^)	Before	0.76 ± 0.26	0.63 ± 0.28	1.02 ± 0.27	0.76 ± 0.24	0.09	0.7705	0.002
	After	0.94 ± 0.30 *	0.74 ± 0.21	1.12 ± 0.24	0.91 ± 0.21 *			
Peak Torque F180 (N·m)	Before	47.08 ± 16.67	34.14 ± 16.70	61.03 ± 27.26	35.13 ± 10.89	0.10	0.7573	0.002
	After	57.86 ± 14.98	36.90 ± 14.05	67.02 ± 20.62	45.16 ± 14.68			
Peak Torque/m F180 (N·m·kg^−1^)	Before	0.56 ± 0.25	0.48 ± 0.24	0.70 ± 0.28	0.56 ± 0.20	0.27	0.6086	0.006
	After	0.70 ± 0.28	0.52 ± 0.23	0.78 ± 0.23	0.71 ± 0.23			

Different then in pre-intervention tests: *—*p* < 0.05. Peak Torque E180—peak torque of knee extension at 180°/s, Peak Torque E180/m—peak torque of knee extension at 180°/s normalized to body mass, Peak Torque F180—peak torque of knee flexion at 180°/s, Peak Torque F180—peak torque of knee flexion at 180°/s normalized to body mass.

**Table 6:** Knee flexors/extensors peak torque ratio at 60°/s and 180°/s.

		GROUP						
		Control		Intervention				
	TESTING SESSION					GROUP × TESTING SESSION		
		Male (n = 8)	Female (n = 12)	Male (n = 15)	Female (n = 11)	F_1,42_	*p*	η^2^
Flexors/Extensors ratio (%) 60	Before	62.20 ± 20.79	56.30 ± 18.10	51.95 ± 10.80	54.82 ± 11.06	3.44	0.0707	0.076
	After	55.08 ± 14.85	56.85 ± 8.75	55.01 ± 8.28	59.90 ± 11.76			
Flexors/Extensors ratio (%) 180	Before	73.16 ± 23.67	73.48 ± 12.36	67.42 ± 15.50	72.91 ± 14.11	0.48	0.4925	0.011
	After	74.29 ± 14.75	68.58 ± 13.87	69.06 ± 13.51	74.49 ± 15.74			

**Table 7:** Functional tests: Time Up and Go (TUG) and Chair Tests pre- and post-intervention.

		GROUP						
		Control		Intervention				
	TESTING SESSION					GROUP × TESTING SESSION		
		Male (n = 8)	Female (n = 12)	Male (n = 15)	Female (n = 11)	F_1,42_	*p*	η^2^
TUG (s)	Before	6.16 ± 0.91	6.83 ± 1.57	5.71 ± 0.89	5.59 ± 0.69	3.06	0.0876	0.068
	After	6.21 ± 1.18	6.65 ± 1.34	5.17 ± 0.63	5.21 ± 0.77			
Chair Test 5STS (s)	Before	9.10 ± 2.56	10.78 ± 4.54	9.48 ± 2.00	8.78 ± 1.98	8.49	0.0057	0.168
	After	8.72 ± 2.47	9.60 ± 3.22	6.65 ± 1.15 ^###^	7.01 ± 1.35 ^#^			
						Z	*p*	R
Chair Test CS-30 (n)	Before	17.5 (13.0–22.5)	19.0 (13.0–19.5)	15.0 (14.0–20.0)	18.0 (14.0–21.0)	4.65	0.0001	0.806
	After	17.0 (14.0–24.0)	16.0 (13.0–19.0)	24.0 (21.0–28.0) *^##^	25.0 (19.0–28.0) **^##^			

Different than Control *—*p* < 0.05, **—*p* < 0.01. Different than in pre- intervention test: ^#^—*p* < 0.05, ^##^—*p* < 0.01, ^###^—*p* < 0.001, n—number of repetitions, s-seconds.

## Data Availability

The datasets generated and analyzed during the current study are not publicly available due to the restrictions involved when obtaining ethical approval for our study, which commit us to share the data only with members of the research team but allow data to be made available from the corresponding author upon reasonable request.
